# EPC-Derived Exosomal miR-1246 and miR-1290 Regulate Phenotypic Changes of Fibroblasts to Endothelial Cells to Exert Protective Effects on Myocardial Infarction by Targeting ELF5 and SP1

**DOI:** 10.3389/fcell.2021.647763

**Published:** 2021-05-13

**Authors:** Yulang Huang, Lifang Chen, Zongming Feng, Weixin Chen, Shaodi Yan, Rongfeng Yang, Jian Xiao, Jiajia Gao, Debao Zhang, Xiao Ke

**Affiliations:** ^1^Departmeng of Cardiology, Shenzhen Nanshan District Shekou People’s Hospital, Shenzhen, China; ^2^Department of Cardiology, Fuwai Hospital, Chinese Academy of Medical Sciences (Shenzhen Sun Yat-sen Cardiovascular Hospital), Shenzhen, China; ^3^Shenzhen University School of Medicine and Shenzhen University Health Science Center, Shenzhen, China

**Keywords:** EPC-derived exosomes, fibroblast-endothelial transition, miR-1246, miR-1290, ELF5, SP1, CD31

## Abstract

Myocardial infarction (MI) remains a leading cause of morbidity and mortality worldwide. Endothelial progenitor cell (EPC)-derived exosomes have been found to be effective in alleviating MI, while the detailed mechanisms remain unclear. The present study aimed to determine the protective effects of EPC-derived exosomal miR-1246 and miR-1290 on MI-induced injury and to explore the underlying molecular mechanisms. The exosomes were extracted from EPCs; gene expression levels were determined by quantitative real-time PCR, and protein expression levels were determined by western blot and immunofluorescence staining, respectively. The angiogenesis and proliferation of human cardiac fibroblasts (HCFs) were determined by tube formation assay and immunofluorescence staining of PKH67, respectively. Luciferase reporter, CHIP, and EMSA assays determined the interaction between miR-1246/1290 and the targeted genes (EFL5 and SP1). The protective effects of miR-1246/1290 on MI were evaluated in a rat model of MI. EPC-derived exosomes significantly upregulated miR-1246 and miR-1290 expression and promoted phenotypic changes of fibroblasts to endothelial cells, angiogenesis, and proliferation in HCFs. Exosomes from EPCs with miR-1246 or miR-1290 mimics transfection promoted phenotypic changes of fibroblasts to endothelial cells and angiogenesis in HCFs, while exosomes from EPCs with miR-1246 or miR-1290 knockdown showed opposite effects in HCFs. Mechanistically, miR-1246 and miR-1290 from EPC-derived exosomes induced upregulation of ELF5 and SP1, respectively, by targeting the promoter regions of corresponding genes. Overexpression of both ELF5 and SP1 enhanced phenotypic changes of fibroblasts to endothelial cells and angiogenesis in HCFs pretreated with exosomes from EPCs with miR-1246 or miR-1290 mimics transfection, while knockdown of both EFL5 and SP1 exerted the opposite effects in HCFs. Both ELF5 and SP1 can bind to the promoter of CD31, leading to the upregulation of CD31 in HCFs. Furthermore, *in vivo* animal studies showed that exosomes from EPCs with miR-1246 or miR-1290 overexpression attenuated the MI-induced cardiac injury in the rats and caused an increase in ELF5, SP1, and CD31 expression, respectively, but suppressed α-SMA expression in the cardiac tissues. In conclusion, our study revealed that miR-1246 and miR-1290 in EPC-derived exosomes enhanced *in vitro* and *in vivo* angiogenesis in MI, and these improvements may be associated with amelioration of cardiac injury and cardiac fibrosis after MI.

## Introduction

Myocardial infarction (MI) remains a leading cause of morbidity and mortality worldwide (Zou et al., 2019). Despite that the progress of effective therapeutics has been made in recent decades, a substantial proportion of MI patients remain to develop congestive heart failure (CHF) (Morice et al., 2010; Izadifar et al., 2014). The necrosis and apoptosis of cardiomyocytes after MI have been recognized as the main contributor for the development of CHF (Laflamme et al., 2002; Mohammad Izadifar et al., 2014). There is growing evidence indicating that restoration of microvascular perfusion in the cardiac tissues may be beneficial for cardiac regeneration and function improvement (Ware and Simons, 1997; Deveza et al., 2012). Cardiac fibroblasts (CFs) are essential for cardiac tissue homeostasis and remodeling, cardiac cell proliferation, and angiogenesis (Furtado et al., 2016). Previous studies have demonstrated that CFs have the potential to transform into pluripotent stem cells, myoblasts, neurons, and endothelial cells (Fan et al., 2012). More importantly, exosomes from endothelial progenitor cells (EPCs) have the potential to promote CFs differentiated into endothelial cells (Ke et al., 2017) by upregulating the expression of mesenchymal–endothelial transition (MEndT)-related genes and increasing the expression of high-mobility group box 1 protein B1 (Ke et al., 2017). However, the regulatory mechanisms underlying the role of CFs in MI remain elusive.

Exosomes, with a diameter of 40–150 nm, are small vesicles secreted by various types of cells. Exosomes contain various molecular constituents of their cell of origin, including proteins and microRNAs (miRNAs), which function as messengers and play important roles in cellular communication. Previous studies have demonstrated that exosomes participate in a variety of physiological and pathological functions such as immune responses and tumor development and relapse (Hough et al., 2017; Oushy et al., 2018; Sundararajan et al., 2018). Notably, one study suggests that mesenchymal stem cell-derived exosomes are beneficial for reducing myocardial ischemia/reperfusion injury (Zhang H. et al., 2016). In addition, exosomes from EPCs were found to regulate the proliferation and migration of endothelial cells *in vitro* (Li X. et al., 2016). However, the mechanisms underlying above biological actions of exosomes are unknown.

MiRNAs belong to a family of newly identified single-strand non-coding RNAs and have 21–23 nucleotides in length. Numerous studies have demonstrated that miRNAs play important roles in diverse cellular processes (e.g., cell proliferation, apoptosis, differentiation) and pathophysiology of various diseases (Chen et al., 2017; Shi et al., 2018). In our preliminary experiments, we performed the microarray analysis and found that miR-1246 and miR-1290 were differentially expressed between EPCs and EPC-derived exosomes from human peripheral blood (Supplementary Table 1). Up to date, miR-1246 and miR-1290 found in exosomes have been suggested to regulate cancer cell proliferation and migration (Sakha et al., 2016; Kobayashi et al., 2018) by targeting the respective downstream mediators (Place et al., 2008; Liu et al., 2013). However, the roles of miR-1246 and miR-1290 in regulating functional processes of CFs have not been examined.

In the present study, we investigated the effects of EPC-derived exosomal miR-1246 and miR-1290 on the phenotypic changes of fibroblasts to endothelial cells, angiogenesis, and proliferation of HCFs by using gain- and loss-of-function studies. Further mechanistic studies were performed to elucidate the downstream targets of EPC-derived exosomal miR-1246 and miR-1290. Finally, the *in vivo* studies were performed to determine the protective effects of EPC-derived exosomal miR-1246 and miR-1290 MI-induced cardiac injury in the rats. The present study may provide novel insights into understanding the role of EPC-derived exosomal miRNAs in pathophysiology of CHFs.

## Materials and Methods

### Cell Isolation and Culture

EPCs were isolated from human peripheral blood as previously described (Ke et al., 2017). In brief, the human blood was collected from healthy volunteers after informed consent was obtained and the current study was approved by the Research Ethic Committee of Fuwai Hospital. The blood samples were diluted with phosphate-buffered saline (PBS) at a 1:1 ratio and added onto a separation medium (GE Healthcare, Pittsburgh, United States) with endothelial cell growth factor and cytokines. Thereafter, the blood sample was centrifuged at 1,200 g for 30 min, and the mononuclear cells were collected and washed with PBS. The cells were then placed into a 25-cm^2^ culture bottle with Endothelial Cell Growth Medium (EGM-2; Thermo Fisher Scientific, Waltham, United States) containing 10% fetal bovine serum (FBS, Gibco, Waltham, United States), 100 U/mL penicillin (Gibco), and 100 μg/mL streptomycin (Gibco). FBS used in this study was centrifuged in advance by density gradient centrifugation in order to remove existing exosomes. After 72 h, non-adherent cells were removed. The medium was refreshed every 3 days, and cells were cultured in a 5% CO_2_-humidified atmosphere at 37°C. Cells at passages 3–6 were used in subsequent experiments.

Human cardiac fibroblasts (HCFs) were purchased from ScienCell Research Lab (San Diego, United States). HCFs were maintained in DMEM/F12 (Thermo Fisher Scientific) containing 10% FBS, 2 mM glutamine, 100 U/mL penicillin, and 100 μg/mL streptomycin at 37°C and 5% CO_2_.

### MiRNAs, siRNAs, Plasmids, and Cell Transfections

The mimics and inhibitors of miR-1246 and -1290 as well as the corresponding negative control (NC) miRNAs were obtained from GenePharma (Shanghai, China). The siRNAs for silencing EFL5 and SP1 and the respective scrambled siRNAs as negative controls were designed and synthesized by GenePharma. The ELF5- and SP1-overexpressing plasmids (pcDNA3.0-ELF5 and pcDNA3.0-SP1) as well as the empty pcDNA3.0 plasmids were purchased from GenePharma. Cell transfections with miRNAs, siRNAs, or plasmids were performed using Lipofectamine 2000 reagent (Invitrogen, Waltham, United States) in accordance with the manufacturer’s protocols.

### Preparation of Exosomes From EPCs

EPCs were cultured in the complete medium until 80% confluence. The medium was replaced with an EGM-2 medium supplemented with 1 × serum replacement solution (PeproTech, Rocky Hill, United States). After incubation for an additional 48 h, the conditioned medium of EPCs was collected and centrifuged at 300 g for 20 min and 2,000 g for 10 min at 4°C in order to remove dead cells and cellular debris. Thereafter, the supernatant was filtered through a 0.22 μm filter (Millipore, ıBurlington, United States), centrifuged at 10,000 g for 30 min. The pellet was discarded, and the supernatant was centrifuged at 100,000 g for 70 min. The pellet was resuspended with phosphate-buffered saline (PBS) and centrifuged at 100,000 g for 70 min and then was resuspended again with PBS and stored at −80°C.

The exosomes were characterized by morphologic examination using a transmission electron microscope (Hitachi H-7650; Japan), and the images were captured with a digital camera (Olympus). Western blots were conducted to detect the proteins of Alix, CD63, and TSG101 in exosomes.

### Immunofluorescence Staining

After different treatments, the HCFs were fixed with 4% PFA, permeabilized with 0.5% Triton X-100, and blocked with 10% goat serum albumin (Invitrogen, Carlsbad, United States) at room temperature. The cells were incubated with primary antibodies including ELF5, CD31, VE-cadherin, VEGFR2, CD31, SP1, and α-SMA at 4°C overnight. Subsequently, the cells were incubated with secondary antibodies at room temperature, and nuclei were counterstained with DAPI. Finally, the immunofluorescence staining was evaluated under a fluorescence microscope (Olympus).

### Flow Cytometry of EPC Surface Markers

EPCs were harvested when approximate 80% confluence was reached, and then incubated at 37°C for 20 min with the following antibodies: CD34-PE (ab157304, 1:45, Abcam), CD133-Alexa Fluor^®^ 488 (#38725, 1:50, Cell Signaling Technology) and VEGFR2-PerCpCy5.5 (ab253080, Abcam, 0.5 μg per reaction). Flow cytometric examination was conducted on FACS Aria III using the soft of FACSDiva 8.0 (BD Biosciences, Franklin Lakes, United States).

### Quantitative Reverse Transcription PCR (qRT-PCR)

A total of RNA was extracted from isolated exosomes, cells, or heart tissues using Trizol Reagent (Takara, Dalian, China) following the manufacturer’s protocols. The concentrations of RNA were determined using a spectrophotometer at 260 nm, and 1 μg of total RNA was reversely transcribed into cDNA using Bestar^TM^ qPCR RT kit (DBI Bioscience, Shanghai, China). Real-time PCR and qPCR reaction was performed on an Applied Biosystems 7500 PCR system (ABI, Foster City, United States) using a Bestar^TM^ qPCR MasterMix kit (DBI Bioscience) under the following conditions: preheating at 95°C for 2 min, followed by 40 cycles of 94°C for 20 s, 58°C for 20 s, and 72°C for 20 s. The primers are presented in Table 1. The expressions of miRNAs were normalized to U6. The expressions of mRNA were normalized to GAPDH. The expression levels were calculated by the 2^–ΔΔCt^ method.

**TABLE 1:** Primers for quantitative real-time PCR.

Name	Primer sequence (5′–3′)
U6 F	CTCGCTTCGGCAGCACA
U6 R	AACGCTTCACGAATTTGCGT
All R	CTCAACTGGTGTCGTGGA
hsa_miR-1246	AAUGGAUUUUUGGAGCAGG
hsa_miR-1246 RT	CTCAACTGGTGTCGTGGAGTCGGCAATTCAGT TGAGCCTGCTCC
hsa_miR-1246 F	ACACTCCAGCTGGGAATGGATTTTTGGAG
hsa_miR-1290	UGGAUUUUUGGAUCAGGGA
hsa_miR-1290 RT	CTCAACTGGTGTCGTGGAGTCGGCAATT CAGTTGAGTCCCTGAT
hsa_miR-1290 F	ACACTCCAGCTGGGTGGATTTTTGGATCA
hsa_miR-375	UUUGUUCGUUCGGCUCGCGUGA
hsa_miR-375 RT	CTCAACTGGTGTCGTGGAGTCGGCAATTC AGTTGAGTCACGCGA
hsa_miR-375 F	ACACTCCAGCTGGGTTTGTTCGTTCGGCTCGC
GAPDH F	TGTTCGTCATGGGTGTGAAC
GAPDH R	ATGGCATGGACTGTGGTCAT
CD31 F	AACAGTGTTGACATGAAGAGCC
CD31 R	TGTAAAACAGCACGTCATCCTT
KDR F	GGCCCAATAATCAGAGTGGCA
KDR R	CCAGTGTCATTTCCGATCACTTT
α-SMA F	CCTGTGTTGTGGTTTACACTGG
α-SMA R	GGGGGAATTATCTTTCCTGGTCC
VE-Cadherin F	GTCAAGGTCAACGTCTTGGAC
VE-Cadherin R	GTTTTATGAGAAGCGTACCAGGT
ELF5 F	TAGGGAACAAGGAATTTTTCGGG
ELF5 R	GTACACTAACCTTCGGTCAACC
SP1 F	TGGCAGCAGTACCAATGGC
SP1 R	CCAGGTAGTCCTGTCAGAACTT

### Enzyme-Linked Immunosorbent Assay (ELISA) for Type I Collagen Detection

The cultured medium of HCFs with different treatments was collected to detect type I collagen using an ELISA kit from Abcam (Cambridge, United States) per manufacturer instructions.

### Western Blot

The exosomes, cells, and tissues collected after treatment were lysed and homogenized with RIPA lysis buffer (Beyotime). After centrifugation at 8,000 g for 10 min, the samples were quantified by BCA method, denatured at 95°C, and loaded at equal amounts onto the SDS-PAGE gels. The proteins were resolved by electrophoresis and then transferred to PVDF membranes (Millipore). Subsequently, the blots were blocked with 5% non-fat milk at room temperature for 1 h and incubated with the primary antibodies including Alix (ab88743, 1:1,000, Abcam), CD63 (ab59479, 1:1,000, Abcam), TSG101 (ab133586, 1:2,000, Abcam), α-SMA (ab32575, 1:3,000, Abcam), CD31 (ab24590, 1:500, Abcam), VE-cadherin (ab166715, 1:1,000, Abcam), VEGFR2 (ab2349, 1:400, Abcam), ELF5 (ab222251, 1:1,000, Abcam), SP1 (ab227383, 1:5,000, Abcam), Lamin A (ab26300, 1:1,000, Abcam) and GAPDH (ab8245, 1:10,000, Abcam) at 4°C overnight. Following incubation with horseradish-peroxidase-conjugated secondary antibodies, the immuno-activities were visualized with the ECL Kit (GE healthcare) and semi-quantified using ImageJ software^1^.

### Cell Phagocytosis Assay

EPCs were incubated with a working solution of acetylated low-density lipoprotein, labeled with 1,1-dioctadecyl-3,3,3,3-tetramethylindocarbocyanine perchlorate (DiL-Ac-LDL; Biomedical Technologies, Tewksbury, United States), at 10 μg/ml. After incubation for 4 h at 37°C, the medium was removed and the cells were fixed in 4% paraformaldehyde (PFA). After incubation with fluorescein isothiocyanate-Ulex europaeus agglutinin-1 (FITC-UEA-l; Sigma-Aldrich, St. Louis, United States) for 1 h at 37°C, the nuclei of cells were counterstained with DAPI (Beyotime, Nantong, China). Finally, the phagocytosis activity was examined and imaged by a fluorescence microscope (Olympus, Tokyo, Japan).

### Tube Formation Assay

After addition with Matrigel (BD, United States), 24-well plates were gently agitated and incubated at 37°C to form a gel. HCFs (4 × 10^6^ cells/well) with different treatments were plated into the coated wells and cultured at 37°C for 30 min. Subsequently, the cells were treated with exosomes (4 μg/ml) of EPCs for 24 h. Tube formation was assessed microscopically, and photographs were captured using an inverted microscope (Olympus).

### Luciferase Reporter Assay

The binding sites between miRNAs and 3′ UTR of the targeted genes were predicted by bioinformatics tools (TargetScan and miRBase). The bioinformatics tool JASPAR was used to predict the transcription factor binding sites. Putative regulatory sequences of CD31, the promoter region of ELF5 and SP1, were synthesized and obtained from GenePharma (Shanghai, China). These fragments were individually cloned into the pGL3-Basic Vector (Cat # E1751, Promega, Madison, United States) to generate the wild-type or mutant type of reporter vectors containing the CD31 promoter, ELF5 promoter, and SP1 promoter, respectively. The reporter vectors were co-transfected into 293T cells with miR-1246 mimics, miR-1290 mimics, pcDNA3.0-ELF5 (OE-ELF5), or pcDNA3.0–SP1 (OE-SP1) or the respective NCs by using Lipofectamine 2000 reagent (Invitrogen, United States) according to the manufacturer’s protocol. At 48 h after co-transfection, luciferase activities were detected using the Dual-Luciferase Report Assay System (Progema).

### Chromatin Immunoprecipitation (CHIP)

The cultured cells were collected and lysed with RIPA lysis buffer. The antibody against CD31 (ab24590; Abcam) or normal IgG (Sigma-Aldrich) as the immunoprecipitation control was applied to Protein A Sepharose beads (GE healthcare) in PBS and incubated at room temperature for 2 h. Following incubation with lysates at 4°C overnight, the protein A Sepharose beads were collected and protein–DNA complexes were eluted. The bound DNA was purified with QIAquick columns (Qiagen, Valencia, United States) and subjected to qRT-PCR assay. Primers are listed in Table 1.

### Electrophoretic Mobility Shift Assay

Nuclear extracts of the cultured cells were prepared using NE-PER Nuclear and Cytoplasmic Extraction Kit (Beyotime). The protein concentration was determined using a BCA method. Double-stranded oligonucleotides labeled with biotin and encompassing a recognized binding element was used as a probe. In brief, nuclear extracts were incubated in binding buffer with the antibodies against ELF5 and SP1 and poly dI:dC in binding buffer on ice for 20 min. Following incubation with a labeled probe at room temperature for 10 min, the DNA–protein complexes were subjected to PAGE electrophoresis and were then transferred to PVDF membrane and cross-linked using an ultraviolet lamp. The membrane was incubated with streptavidin–HRP conjugate at room temperature for 15 min and visualized by the ECL kit (GE healthcare).

### Myocardial Infarction (MI) Model Establishment

Male Sprague-Dawley (SD) rats weighting about 200 g were obtained from the Animal Center of Southern Medical University and housed in specific pathogen-free conditions with a temperature of 20–26°C and a relative humidity of 40–75% with a light cycle of 12-h light/12-h dark. Myocardial infarction (MI) was induced by ligation of the left anterior descending coronary artery. In brief, the rats were anesthetized by intraperitoneal injection of pentobarbital sodium (40 mg/kg; Sigma). After left thoracotomy, the heart was exposed and the left anterior descending artery was occluded using a 6–0 monofilament nylon suture. With electrocardiographic evidence of acute myocardial infarction, the heart was placed back to the chest and the thorax was closed and sutured. A sham group (10 rats) was established by the same operation without coronary artery ligation. The MI rats were randomly assigned into four groups (10 rats/group). For the model group, 200 μL PBS was injected into the border zone of the infarcted area; for the NC/Exo group, the exosomes from mimics NC-transfected EPCs were injected into the border zone of the infarcted area (100 μg in 200 μL PBS); for the Mimics-1/Exo group, the exosomes from miR-1246 mimics-transfected EPCs were injected into the border zone of the infarcted area (100 μg in 200 μL PBS); and for the Mimics-2/Exo group, the exosomes from miR-1290 mimics-transfected EPCs were injected into the border zone of the infarcted area (100 μg in 200 μL PBS). At 8 weeks after different treatments, all the rats were subjected to echocardiography examination before the rats were killed by intraperitoneal injection of pentobarbital sodium (80 mg/kg). After that, the hearts were collected for 2,3,5-triphenyltetrazolium (TTC) staining, hematoxylin and eosin (H&E) staining, Masson staining, immunohistochemical staining, qRT-PCR, and western blot analysis.

### Histological Examination

The heart tissues fixed in 10% formaldehyde were dissected, dehydrated by a series of ethanol solutions with ascending concentrations, immersed in xylene, and then embedded in paraffin. The sections were prepared, de-waxed with xylene, and rehydrated with ethanol solutions with descending concentrations. Subsequently, the sections were stained with H&E (Servicebio, Wuhan, China) or Masson’s trichrome (Servicebio) staining for histological assessment.

To observe the infarct injury, the hearts were rinsed with sterile PBS and frozen in a freezer until stiffness. Subsequently, the hearts were cut into 3-mm sections from apex to bottom. The sections were immersed in pre-warmed 2% TTC (Sigma-Aldrich) for 30 min at 37°C and then fixed with 10% formaldehyde for morphological examination.

### Immunohistochemistry

Heart tissues were fixed in 4% formaldehyde and embedded in paraffin; 5 μm-thick slices were sectioned. The slides were permeabilized with 1% Triton X-100 in PBS for 30 min at room temperature, boiled in 100 mM sodium citrate pH 6.0 three times for 6 min each at 5 min intervals for antigen retrieval, and then washed with 3% hydrogen peroxide for 30 min to remove endogenous peroxidase. After being blocked with 10% bovine serum albumin, the sections were incubated with primary antibodies against ELF5, SP1, CD31, and α-SMA at 4°C overnight. Subsequently, the samples were incubated with a secondary antibody at room temperature for 2 h. Immuno-reactivity was visualized by using 3,3-diaminobenzidine chromogen. The sections were counterstained with hematoxylin and examined under a light microscope (Olympus).

### Statistical Analysis

The statistical analysis of all the datasets was conducted by the SPSS 17.0 software (IBM, Armonk, United States). Data are presented as mean ± standard deviation. Significant differences among different treatment groups were assessed by one-way analysis of variance followed by Bonferroni’s *post hoc* test. *P-*values < 0.05 were considered as statistically significant.

## Results

### Identification of EPCs and Exosomes

**FIGURE 1:**
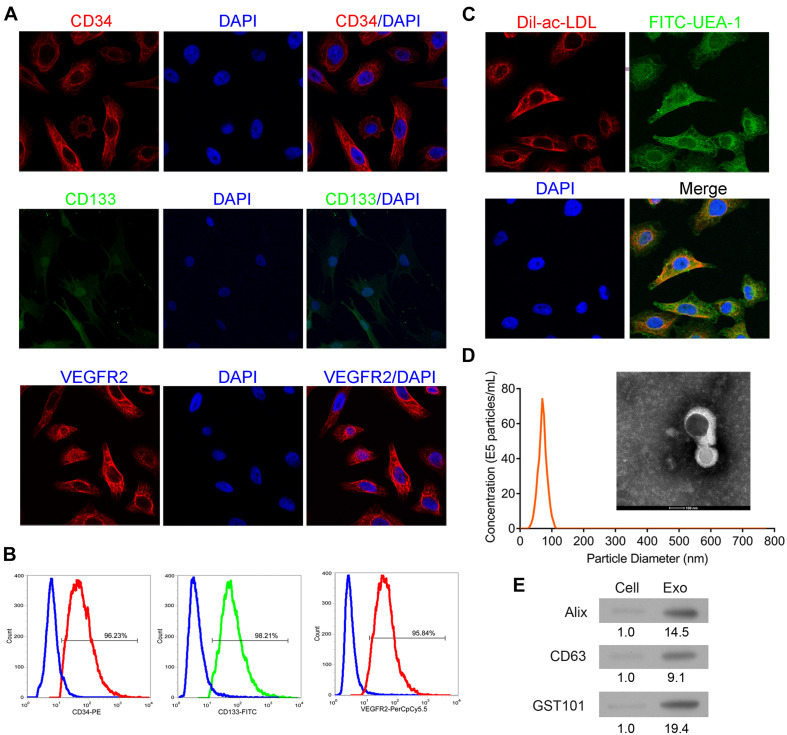
Identification of EPCs from human peripheral blood and exosomes from EPCs. The isolated cells were cultured and then collected for detection of CD34, CD133, and VEGFR2 expressions by immunofluorescence staining **(A)** and flow cytometry **(B)**. Additionally, the phagocytic ability of the cells was examined by dual immuno-staining of Dil-Ac-LDL and FITC-UEA-1 **(C)**. The morphology, size, and concentration of the isolated exosomes were identified by transmission electron microscope and Nanosight tracking analysis, scale bar = 100 nm **(D)**. The expressions of exosome-associated proteins, including Alix, CD63, and TSG101 were detected by western blot **(E)**. EPCs, endothelial progenitor cells. *N* = 3.

EPCs were confirmed by examination of biomarkers and phagocytosis activity. As shown in Figures 1A,B, the isolated cells were positive for endothelial cell makers including CD34, CD133, and VEGFR2. The cells demonstrated the activity of phagocytosis (Figure 1C). These results indicated that the isolated cells were EPCs. As shown in Figure 1D, exosomes exhibited a round morphology with a diameter approximately between 40 and 120 nm. Western blot analysis further detected the protein expressions of Alix, CD63, and TSG101 in the isolated vesicles (Figure 1E). These results implied that exosomes were successfully extracted.

### EPC-Derived Exosomes Promoted Phenotypic Changes of Fibroblasts to Endothelial Cells, Angiogenesis, and Proliferation in HCFs

**FIGURE 2:**
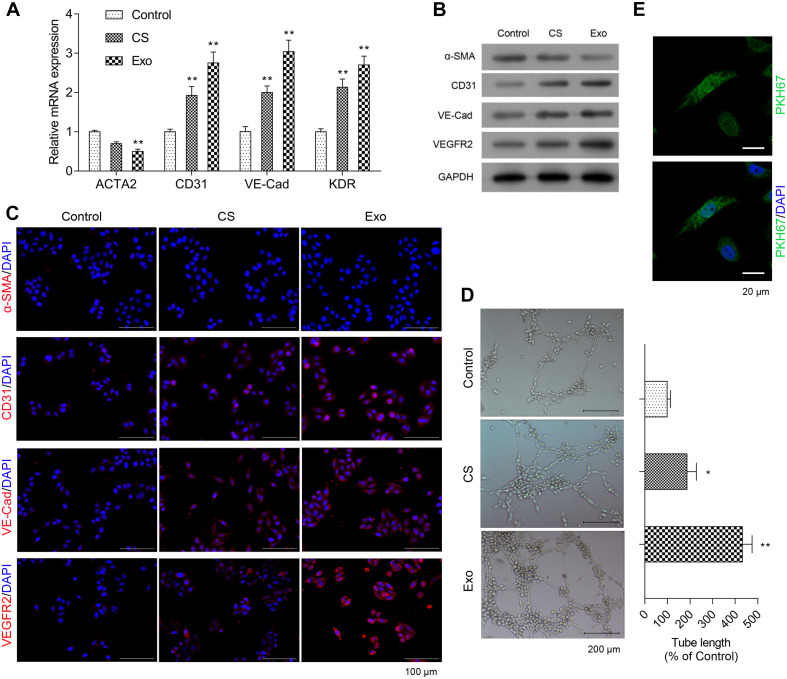
Exosomes from EPCS enhanced angiogenesis and proliferation of HCFs. In the HCFs treated with control (PBS), cell supernatant (CS), or exosomes from EPCs (Exo), the transcriptional expressions of ACTA2, CD31, VE-Cadherin (VE-Cad), and VEGFR2 were determined by qRT-PCR **(A)**, and the expressions of α-SMA, CD31, VE-Cad, and VEGFR2 were determined by western blot **(B)** and immunofluorescence staining **(C)**. Additionally, the angiogenesis was examined by tube formation assay **(D)**, and the cell proliferation was determined by a fluorescent probe, PKH67 **(E)**. The cells were treated with exosomes (4 μg/ml) of EPCs for 24 h. *N* = 3. *P < 0.05 and **P < 0.01 vs. control group. HCFs, human cardiac fibroblasts.

To determine the effect of EPC-derived exosomes on phenotypic changes of fibroblasts to endothelial cells, HCFs and endothelial cell markers were assessed. As shown in Figure 2A, the mRNA expression level of ACAT2 in HCFs was significantly decreased when exposed to exosomes, while CD31, VE-Cadherin, and KDR in HCFs were significantly increased. Consistently, the western blot and immunofluorescence staining results showed that the significantly downregulated protein expression of α-SMA and upregulated protein expression of CD31, VE-Cadherin, and VEGFR2 in HCFs with EPC-derived exosomes were observed (Figures 2B,C and Supplementary Figure 1). Angiogenic activity of the cells was determined by tube formation assay. As shown in Figure 2D, exosomes significantly induced angiogenesis of HCFs, suggesting that EPC-derived exosomes might promote fibroblast-endothelial transition of HCFs. Moreover, the cell proliferative ability of HCFs was significantly enhanced after exposure to the EPC-derived exosomes, as reflection by the significantly increased expressions of PKH67 (Figure 2E).

### The Roles of miR-1246 and miR-1290 in EPC-Derived Exosome-Induced Phenotypic Changes of Fibroblasts to Endothelial Cells

**FIGURE 3:**
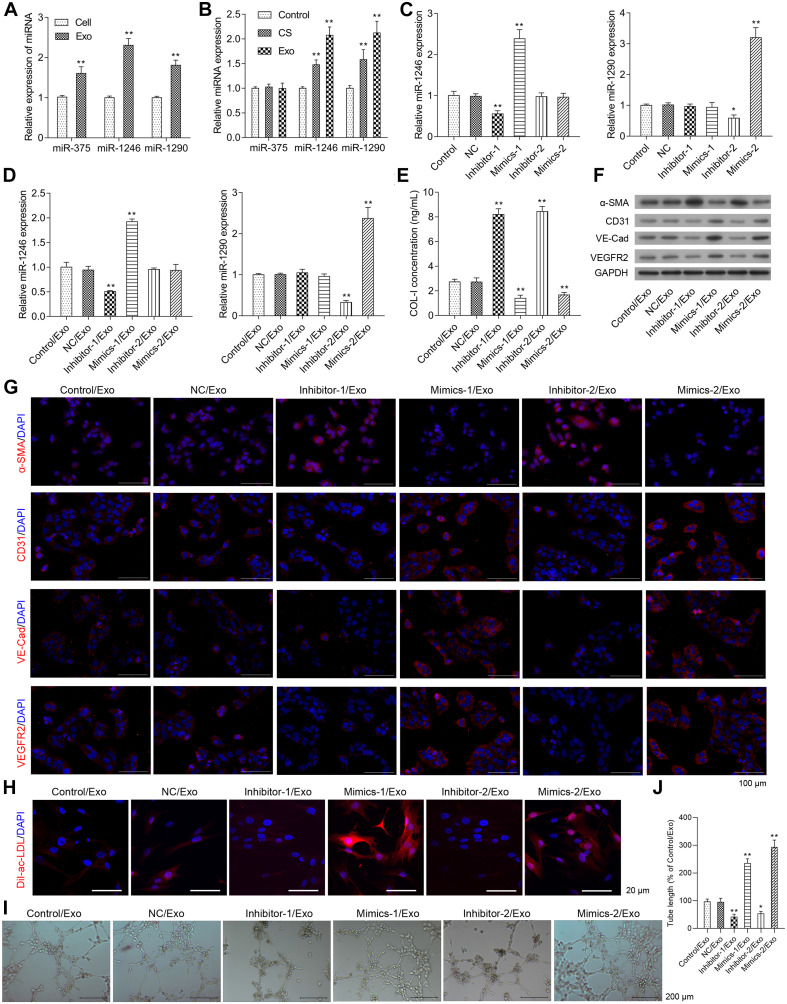
Effects of miR-1246 and miR-1290 on angiogenesis induced by exosomes from EPCs. The miR-375, miR-1246, and miR-1290 levels in exosomes itself or in whole EPCs were determined via real-time PCR **(A)**. After treatment with control, CS, or Exo, HCFs were harvested to determine the expressions of miR-375, miR-1246, and miR-1290 **(B)**. The EPCs received one of the following transfections: miR-1246 inhibitor-1, miR-1246 mimics-1, miR-1290 inhibitor-2, Mimics-2, and NCs. Then, the expressions of miR-1246 and miR-1290 in exosomes derived from the parent or transfected EPCs were determined **(C)**. HCFs were treated with exosomes from parent EPCs (Con/Exo), exosomes from EPCs transfected with NC (NC/Exo), miR-1246 inhibitor-1 (inhibitor-1/Exo), miR-1246 mimics-1 (Mimics-1/Exo), miR-1290 inhibitor-2 (inhibitor-2/Exo), or miR-1290 mimics-2 (Mimics-2/Exo). Then, the cells were collected, expressions of miR-1246 and miR-1290 were determined by qRT-PCR **(D)**, the level of Collagen-I (Col-I) in the medium was determined by ELISA **(E)**, and the expressions of α-SMA, CD31, VE-Cad, and VEGFR2 were determined by western blot **(F)** and immunofluorescence staining **(G)**. Additionally, the phagocytic activity of HCFs was determined by DiL-Ac-LDL staining **(H)**, and the angiogenesis was measured by a method of tube formation assay **(I)**. The tube length in each group was quantified according to the results of tube formation assay **(J)**. The cells were treated with exosomes (4 μg/ml) of EPCs for 24 h. *N* = 3. *P < 0.05, **P < 0.01 vs. NC/Exo group. Exo, exosomes; Mimics-1, miR-1246 mimics; Mimics-2, miR-1290 mimics; Inhibitor-1, miR-1246 inhibitor; Inhibitor-2, miR-1290 inhibitor.

The miR-375, 1246, and 1290 levels were higher in exosomes isolated from EPCs than those in whole EPCs (Figure 3A). As shown in Figure 3B, EPC-derived exosomes had no effect on the expression of miR-375 in HCFs when compared to the control group, while miR-1246 and miR-1290 expressions were significantly increased in HCFs treated with culture supernatant and EPC-derived exosomes, and the expression levels of miR-1246 and miR-1290 in HCFs treated with EPC-derived exosomes were much higher than that in culture supernatant-treated HCFs (Figure 3A).

As shown in Figure 3C, miR-1246 mimics (Mimics-1) and miR-1290 mimics (Mimics-2) transfection significantly increased miR-1246 and miR-1290 expression levels in EPC-derived exosomes, respectively, while miR-1246 inhibitor (inhibitor-1) and miR-1290 inhibitor (inhibitor-2), respectively downregulated miR-1246 and miR-1290 expression in EPC-derived exosomes. The expressions of miR-1246 and miR-1290 were significantly upregulated in HCFs with the respective treatment by exosomes derived from miR-1246 mimics- and miR-1246 mimics-transfected EPCs, while the expressions of miR-1246 and miR-1290 were significantly downregulated in HCFs with the respective treatment by exosomes derived from miR-1246 inhibitor- and miR-1246 inhibitor-transfected EPCs. Exosomes from miR mimics or inhibitors increased or decreased the expressions of miR-1246 and -1290 in CFs (Figure 3D).

Collagen-I expressions in the HCF medium and α-SMA expressions in HCFs were significantly increased in the Inhibitor-1/Exo group, while they were significantly decreased in the Mimics-1/Exo and Mimics-2/Exo groups (Figures 3E–G and Supplementary Figure 2A). In contrast, protein expressions of CD31, VE-Cadherin, and VEGFR2 in HCFs were decreased in the Inhibitor-1/Exo and Inhibitor-2/Exo groups, while increased expressions of CD31, VE-Cadherin, and VEGFR2 in HCFs were observed in Mimics-1/Exo and Mimics-2/Exo groups (Figures 3F,G and Supplementary Figure 2A). Similarly, the phagocytic and angiogenic activities of HCFs were significantly increased in the Mimics-1/Exo and Mimics-2/Exo groups (Figures 3H–J and Supplementary Figure 2B) and were decreased in the Inhibitor-1/Exo and Inhibitor-2/Exo groups.

### MiR-1246 and MiR-1290 Induced ELF5 and SP1 Expressions

**FIGURE 4:**
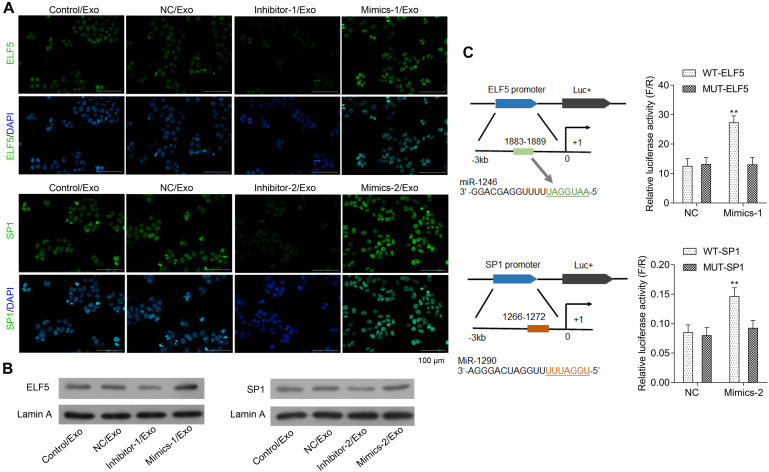
Interaction between miR-1246 and ELF5, miR-1290, and SP1. In HCFs treated with Con/Exo, NC/Exo, inhibitor-1/Exo, Mimics-1/Exo, inhibitor-2/Exo, or Mimics-2/Exo, the expressions of ELF5 and SP1 were determined by immunofluorescence staining **(A)** and by western blot together with Lamin A **(B)**. Potential binding of miR-1246 to the promoter of ELF-5 and miR-1290 to that of SP1 and induced expressions of the genes were observed in luciferase report assay **(C)**. *N* = 3. **P < 0.01 vs. NC group. Exo, exosomes; Mimics-1, miR-1246 mimics; Mimics-2, miR-1290 mimics; Inhibitor-1, miR-1246 inhibitor; Inhibitor-2, miR-1290 inhibitor.

The bioinformatics analysis showed that ELF5 and SP1 might be the most potential target genes of miR-1246 and miR-1290. As shown in Figures 4A,B, expressions of ELF5 and SP1 were significantly upregulated in HCFs in Mimics-1/Exo and Mimics-2/Exo groups, respectively, but were significantly downregulated in HCFs in Inhibitor-1/Exo and Inhibitor-2/Exo groups, respectively (Supplementary Figure 3). The luciferase reporter assay showed that miR-1246 and miR-1290 directly interacted with the promoters of ELF5 and SP1, respectively, resulting in increased expressions of ELF5 and SP1, respectively (Figure 4C).

### Role of ELF5 and SP1 in Regulating Phenotypic Changes of Fibroblasts to Endothelial Cells

**FIGURE 5:**
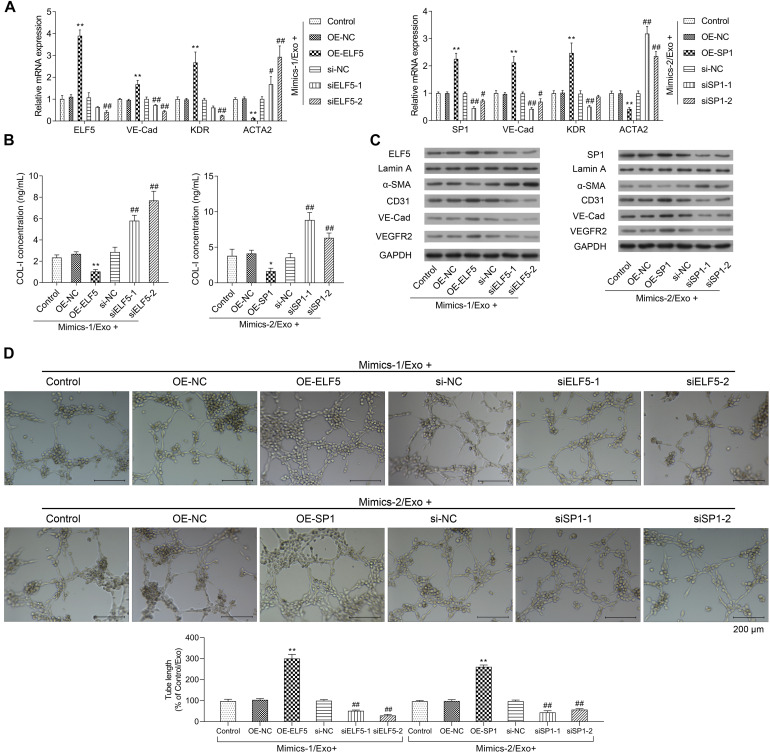
Role of ELF5 and SP1 in angiogenesis induced by exosomes from EPCs. In parent HCFs or those transfected with empty plasmids (OE-NC), SP1 overexpression plasmids (OE-SP1), ELF5 overexpression plasmids (OE-ELF5), siRNA-1 against SP1 (siSP1-1), siRNA-2 against SP1 (siSP1-2), siRNA-1 against ELF5 (siELF5-1), siRNA-2 against ELF5 (siELF5-2), or the negative control (si-NC), the expressions of ELF5, SP1, VE-Cad, VEGFR2, and ACTA2 were determined by qRT-PCR **(A)**, the level of COL-I in medium was examined by ELISA **(B)**, and the proteins levels of ELF5, SP1, Lamin A, α-SMA, CD31, VE-Cad, and VEGFR2 were determined by western blot **(C)**. Additionally, the angiogenesis of the cells were determined by tube formation assay **(D)**. *N* = 3. *P < 0.05, **P < 0.01 vs. OE-NC group; ^#^*P* < 0.05, ^##^*P* < 0.01 vs. si-NC group. Exo, exosomes; Mimics-1, miR-1246 mimics; Mimics-2, miR-1290 mimics.

Moreover, the bioinformatics tool JASPAR was applied to predict the transcription factor binding sites, and the data showed that both ELF5 and SP1 bound to the promoter of CD31 (see Table 2 for the predicted binding sites). After treatment with Mimics-Exo, mRNA levels of ELF5 and SP1 were significantly increased in HCFs transfected with ELF5 and SP1 overexpression plasmids, while those transfected with ELF5 and SP1 siRNAs were decreased (Figures 5A,B). Similarly, mRNA levels of VE-Cadherin and KDR were significantly increased and those of ACTA2 were significantly decreased in HCFs with ELF5 or SP1-overexpressing plasmids, and the opposite effects were observed in HCFs transfected with corresponding siRNAs (Figures 5A,B). Consistent results were further revealed by examining the protein levels of ELF5, SP1, α-SMA, CD31, VE-Cad, and VEGFR2 using western blot in HCFs (Figure 5C). In the HCFs treated with Mimics-1/Exo or Mimics-2/Exo, ELF5 and SP1 overexpression both significantly enhanced tube formation of HCFs, while ELF5 and SP1 knockdown exerted the opposite effects (Figure 5D). Collectively, these results suggest that ELF5 and SP1 might have played important roles in HCFs transforming into endothelial cells and subsequent angiogenesis.

**TABLE 2:** The predicted binding sites between SP1/ELF5 and CD31.

Model name	CD31 promoter region			
	
	Start	End	Strand	Predicted site sequence
SP1	1,756	1,766	1	CCTCCGCCTCC
ELF5	2,567	2,575	1	TACTTCCTC

### Interaction Between ELF5 and CD31, SP1, and CD31

**FIGURE 6:**
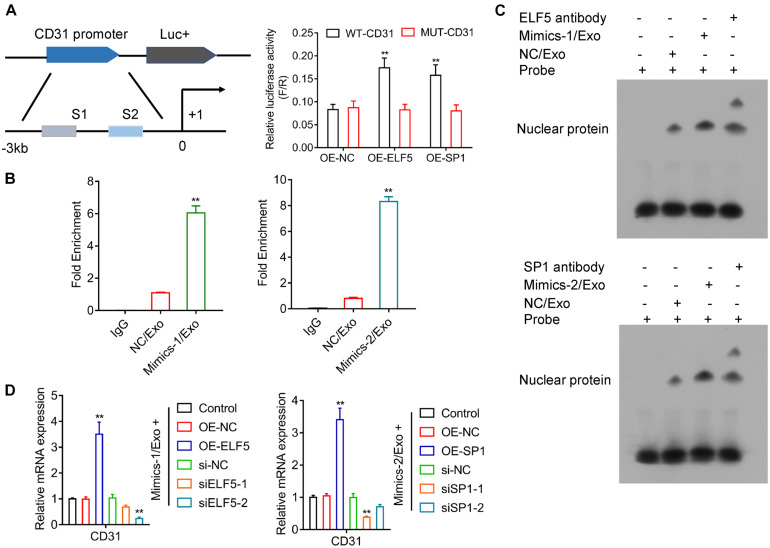
Regulation of CD31 directly by both ELF5 and SP1. Potential binding of ELF5 and SP1 to the promoter of CD31 and induction of the expression were examined by luciferase report assay **(A)**. In HCF cells treated with control (IgG), NC/Exo, mMimics-1/Exo or Mimics-2/Exo, the association of miR-1246 and miR-1290 with the potential binding of ELF5 and SP1 to the DNA of CD31, respectively, was examined by CHIP **(B)** and EMSA **(C)**. In HCFs that received non-transfection or transfection with OE-NC, OE-ELF, or OE-SP1 (si-NC, siELF5-1, siELF5-2, siSP1-1, or siSP1-2), the expression of CD31 was examined by qRT-PCR **(D)**. *N* = 3. **P < 0.01 as compared with the NC group. Exo, exosomes; Mimics-1, miR-1246 mimics; Mimics-2, miR-1290 mimics.

Luciferase reporter assay showed that both ELF5 and SP1 bound to the promoters of CD31, as indicated by increased luciferase activity of the reporter vector containing the wild-type promoter region of CD31 (Figure 6A), suggesting that CD31 expression was induced by ELF5 and SP1 via binding to its promoter. The CHIP and EMSA assay showed that the binding between ELF5 and CD31 was enhanced in the Mimics-1/Exo group, and the binding between SP1 and CD31 was enhanced in the Mimics-2/Exo group (Figures 6B,C and Supplementary Figure 4). The effects of ELF5 and SP1 on CD31 expression were further examined in HCFs with ELF5 and SP1 overexpression or knockdown, respectively. Both ELF5 and SP1 overexpression significantly increased CD31 expression, whereas ELF5 and SP1 knockdown decreased CD31 expression in HCFs with treated Mimics-1/Exo or Mimics2/Ex0 (Figure 6D), suggesting that both ELF 5 and SP1 played important roles in exosome-induced CD31 expression.

### Effects of miR-1246 and miR-1290 on MI in Rats

**FIGURE 7:**
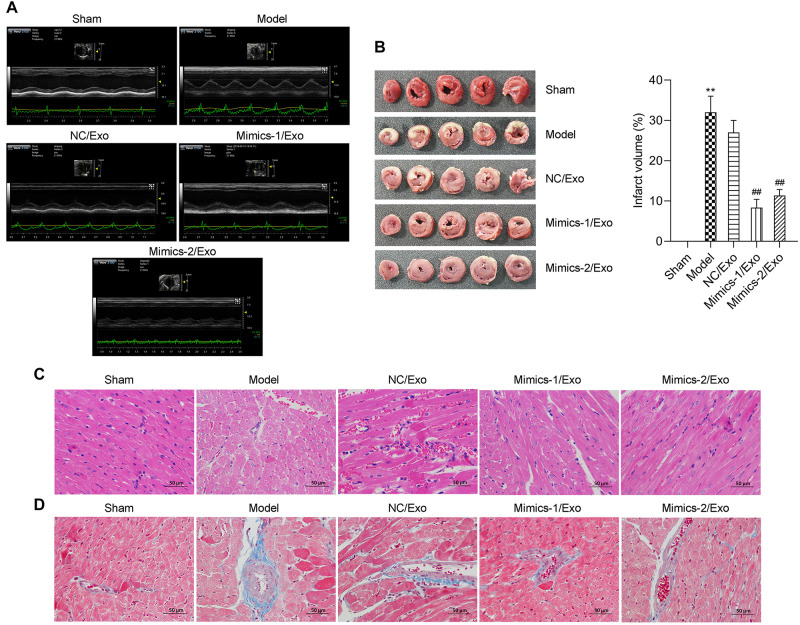
Effects of miR-1246 and miR-1290 in exosomes from EPCs on myocardial infarction in rats. After administration of PBS to sham and model groups, and of exosomes from EPCs transfected with NC/Exo, Mimics-1/Exo, or mMimics-2/Exo, cardiac function was evaluated by echocardiography **(A)**. Myocardial infarction was evaluated on infarction size through TTC, the infarct volume was measured, and the infarct volume percentage was calculated **(B)**. The histopathological feature was identified through H&E staining **(C)**. The degree of tissue fibrosis was determined through Masson staining **(D)**. *N* = 10. **P < 0.01 as compared with the Sham group; ^##^*P* < 0.01 as compared to the Model group. Exo, exosomes; Mimics-1, miR-1246 mimics; Mimics-2, miR-1290 mimics.

As shown in Table 3, compared to the sham group, heart rate (HR), diastolic left ventricular volume (Vd), stroke volume (SV), left ventricular ejection fraction (LVEF) fractional shortening (FS), and cardiac output (CO) were significantly lowered, and the left ventricular end systolic diameter (LVESd) was markedly elevated in the model group when compared to the sham group, while the changes in these indicators from the model group could be significantly reversed by in the Mimics-1/Exo and Mimics-2/Exo groups (Figure 7A and Table 3). TTC staining results exhibited that the infarct volume was dramatically increased in the model group when compared to the sham group (Figure 7B), while the increase in infarct volume could also be markedly reversed in the Mimics-1/Exo and Mimics-2/Exo groups (Figure 7B). Additionally, H&E staining results disclosed that in the sham group, the myocardial fibers were arranged regularly and had a complete structure; in the model group, the disordered cardiac structure was evident, such as wave-like changes of rough myofilaments, interstitial congestion, endothelial disruption, and cardiomyocytes lysis; and in the Mimics-1/Exo and Mimics-2/Exo groups, the disordered cardiac structure was dramatically improved (Figure 7C). Masson staining results showed that the fibrosis was dramatically enhanced in the model group compared with the sham group, which was significantly reversed in the Mimics-1/Exo and Mimics-2/Ex groups (Figure 7D).

**TABLE 3:** The related indicators of cardiac function in echocardiography.

Indicators	HR	LVESd (mm)	LVEDd (mm)	Vs (μ L)	Vd (μ L)	SV (μ L)	LVEF (%)	FS (%)	CO (mL/min)	LVPWT (mm)	LVAWT (mm)
Sham	35411.53	3.70.18	6.140.32	101.064.85	246.028.42	146.165.45	74.741.48	35.481.2	50.792	2.230.09	2.070.11
Model	281.339.61**	5.380.22**	6.820.46	98.616.58	211.695.96**	115.913.42**	51.172.07**	26.852.19**	34.012.17**	2.170.08	1.920.1
NC-Exo	303.6712.7	4.890.21	6.620.38	96.472.94	213.38.42	120.474.99	53.442.93	28.762	37.281.86	2.340.18	2.10.13
Mimics-1/Exo	323.673.21^##^	4.540.24^##^	6.710.3	100.172.5	229.772.1^#^	131.543.03^##^	60.452.07^##^	32.580.95^##^	43.743.29^##^	2.270.07	1.960.09
Mimics-2/Exo	327.6711.02^##^	4.120.28^##^	6.620.27	97.943.47	237.574.53^##^	136.862.35	65.172.54^##^	34.310.79^##^	45.392.55^##^	2.150.07	2.070.08

**P < 0.01 vs. sham group, ^#^P < 0.05, ^##^P < 0.01 vs. model group. Exo, exosomes; Mimics-1, miR-1246 mimics; Mimics-2, miR-1290 mimics; Inhibitor-1, miR-1246 inhibitors; Inhibitor-2, miR-1290 inhibitors; HR, heart rate; LVESd, left ventricular end systolic diameter; LVEDd, left ventricular end-diastolic diameter; Vs, systolic left ventricular volume; Vd, diastolic left ventricular volume; SV, stroke volume; LVEF, left ventricular ejection fraction; FS, fractional shortening; CO, cardiac output; LVPWT, left ventricular posterior wall thickness; LVAWT, left ventricular diastolic anterior wall thickness.

**FIGURE 8:**
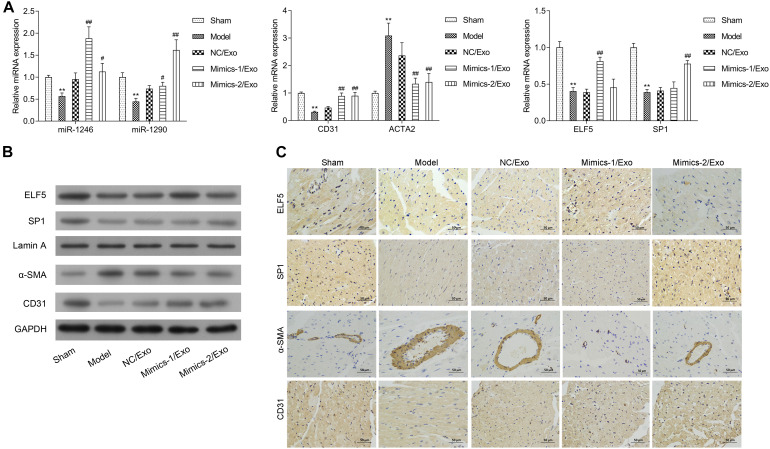
Effects of miR-1246 and miR-1290 in exosomes from EPCs on angiogenesis in rats with myocardial infarction. After administration of PBS to sham and model groups, and of exosomes from EPCs transfected with NC/Exo, Mimics-1/Exo, or Mimics-2/Exo, the expressions of miR-1246, miR-1290, CD31, ACTA2, ELF5, and SP1 were examined by qRT-PCR **(A)**, the expressions of ELF5, SP1, Lamin A, α-SMA, and CD31 were examined by western blot **(B)**, and the expressions of ELF5, SP1, α-SMA, and CD31 were also examined by IHC **(C)**. *N* = 10. **P < 0.01 as compared with the Sham group; ^#^*P* < 0.05, ^##^*P* < 0.01 as compared with the Model group. Exo, exosomes; Mimics-1, miR-1246 mimics; Mimics-2, miR-1290 mimics.

The expression of miR-1246 and miR-1290 in cardiac tissues was significantly downregulated in the model group when compared to the sham group (Figure 8A). The expressions of miR-1246 and miR-1290 were significantly increased in cardiac tissues after treatment with Mimics-1/Exo and Mimics-2/Exo, respectively (Figure 8A). ELF5 and SP1 expressions were also significantly increased after treatment with Mimics-1/Exo and Mimics-2/Exo, respectively (Figure 8A). Meanwhile, an increase in CD31 and a decrease in ACTA2 mRNA levels were observed after treatments with Mimics-1/Exo and with Mimics-2/Exo (Figure 8A). Subsequently, the western blotting results uncovered that ELF5, SP1, and CD31 expressions were downregulated, and α-SMA expression was elevated in the model group when compared to the sham group, while the treatments of Mimics-1/Exo or Mimic-2/Exo could markedly upregulate ELF5, SP1, and CD31 and downregulate α-SMA in the model rats (Figure 8B). Moreover, the immunohistochemical results further confirmed that the treatments of Mimics-1/Exo or Mimic-2/Exo significantly decreased α-SMA expression and increased ELF5, SP1, and CD31 expressions in the model rats (Figure 8C and Supplementary Figure 5).

## Discussion

**FIGURE 9:**
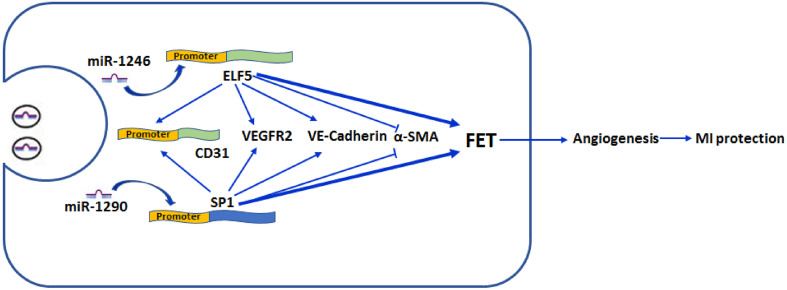
Schematic illustration/of effect by exosomal mir-1246 and -1290 on myocardial infarction. MiR-1246 and -1290 in EPCs could bind to the promoter of ELF5 and SP1 in HCFs, respectively, and induce their expressions. Resultantly, increased ELF5 and SP1 promoted fibroblast-endothelial transition, demonstrated by increased expressions of endothelial markers, CD31 (by binding to the promoter), VEGFR2, and VE-Cad, and decreased expression of a fibroblast marker, α-SMA. Furthermore, they increased angiogenesis. In rats, both exosomal miR-1246 and -1290 contribute to the exosome-attenuated myocardial infarction (MI). All these indicated that both miR-1246 and -1290 in exosomes from EPCs protected the heart against MI, and this was potentially through increased expressions of ELF5 and SP1.

A substantial proportion of patients will develop cardiac dysfunctions after MI (Talman and Ruskoaho, 2016). Previous studies suggest that angiogenesis is beneficial for cardiac function improvement, which has been recognized as a promising therapeutic strategy (Cochain et al., 2013). In the present study, we for the first time showed that EPC-derived exosomes were beneficial for ameliorating ischemia-induced cardiac injury *in vivo.* In addition, our study also showed that exosomes promoted HCFs transforming into endothelium and angiogenesis. Increased expressions of ELF5 and SP1 by miR-1246 and miR-1290 may contribute to protective effects of EPC-derived exosomes on MI-induced cardiac injury (Figure 9).

EPCs circulate in peripheral blood and play important roles in regulating the activities and functions of other cells through excreting exosomes and other paracrine factors. Previous studies have demonstrated that EPCs promoted angiogenesis in injured tissues, which made EPC transplantation an attractive approach for tissue repair. However, EPC transplantation has several limitations, including embolization and malignant transformation (Herberts et al., 2011; Jia et al., 2019). Accumulating evidence indicated that EPC-derived exosomes exerted similar functions as EPCs. Consistent with previous reports (Zhang J. et al., 2016; Sun et al., 2018; Zhou et al., 2018), our results showed that EPC-derived exosomes were capable of promoting angiogenesis in cardiac tissues after ischemia injury, suggesting that EPC-derived exosomes might substitute EPCs for tissue repair.

Several miRNAs in exosomes are associated with angiogenesis (Umezu et al., 2014; Hu et al., 2018). For example, Ma et al. reported that miR-210 in EPC-derived exosomes induced angiogenesis under hypoxia/reperfusion injury and the mechanisms underlying these processes were due to protect endothelial functions and improve mitochondrial function (Ma et al., 2018). Two studies have demonstrated that miR-216 in EPC derived exosomes promoted angiogenesis by means of activating the Ras/ERK signaling pathway (Wu et al., 2018; Jia et al., 2019). Bas et al. reported that miR-214 in endothelium-derived exosomes enhanced angiogenesis by stimulating endothelial cell migration and proliferation (van Balkom et al., 2013). MiRNA, namely, let-7f-5p, is transferred by exosomes to endothelial cells, which in turn promoted vascular repair (Ribeiro et al., 2013). As exosomal miR-375 has been implicated for its role in pathophysiology of various diseases (Huang et al., 2015; Chen et al., 2019; Meltzer et al., 2019; Li et al., 2020; Zhang et al., 2020), we also detected the expression level of miR-375 after EPC-exosome treatment and found that the miR-375 expression level was not affected, suggesting that miR-375 may not be involved in the EPC-exosome-mediated effects. On the other hand, our further results demonstrated that both miR-1246 and miR-1290 in EPC-derived exosomes increased angiogenesis in cardiac tissues, and the mechanisms underlying these benefits were related to enhancement of CFs transforming into endothelial cells. EPC-derived exosomes inducing CF differentiation into endothelial cells also supports our current findings (Ke et al., 2017). In addition, the pro-angiogenic effects of EPC-derived exosomes might also be associated with Erk1/2 signaling activation, which in turn increased the expressions of angiogenesis-related molecules including FGF-1, VEGFA, VEGFR-2, ANG-1, E-selectin, CXCL-16, and eNOS (Li X. et al., 2016; Zhang J. et al., 2016).

ELF5 and SP1 are well-known transcriptional factors, which play important roles in multiple essential biological processes including cell proliferation, angiogenesis, and metastasis (Fukuda et al., 2004; Latos et al., 2015; Su et al., 2017). ELF5 is an epithelial-specific member of the Ets transcription factor family and regulates a variety of cellular biological processes including keratinocyte terminal differentiation, trophoblast differentiation, and epithelial–mesenchymal transformation in tumor cells (Xu et al., 2017). Besides, ELF5 could promote angiogenesis by recruiting myeloid-derived suppressor cells (Gallego-Ortega et al., 2015). Furthermore, ELF5 was essential for progenitor cells differentiating into differentiated endothelial cells, which is potentiated by ELF5-mediated notch signaling activation (Omata et al., 2018). SP-1 is a nuclear transcription factor and regulates tumor proliferation, migration, angiogenesis, and invasion (Vizcaino et al., 2015). SP-1 is also involved in the occurrence and development of cardiovascular diseases and plays an important regulatory role in various pathophysiological processes such as myocardial cell apoptosis, myocardial fibrosis, inflammation, and vascular calcification (Schüttler et al., 2019). Li et al. found that miR-7a/b could improve cardiac function and inhibit MF and apoptosis after myocardial infarction mainly by regulating SP-1 and POLYadenosine diphosphate ribose polymerase-1 in a myocardial infarction mouse model (Li R. et al., 2016). Moreover, the angiogenic activity of SP1 might be associated with increased VEGF expression (Su et al., 2017). However, the roles of ELF5 and SP1 in MI are still unknown. In our study, we showed that miR-1246 and miR-1290 bound to the promoters of ELF5 and SP1, resulting in the increase of ELF5 and SP1 expressions. The current study also for the first time confirmed that both ELF5 and SP1 bound to the promoter of CD31, leading to the increased expressions of CD31 and angiogenesis (Aroca et al., 1999).

There are several limitations that should be considered in the present study. Firstly, the characterization of the fibroblast and endothelial cells has been limited to certain markers; for example, CD31 expression does not induce endothelial differentiation but is expressed by terminally differentiated cells, and future studies should use more markers to confirm the phenotype changes of the cells. Secondly, the tube formation assay may be performed with LDL uptake and lectin staining, which may further confirm the angiogenic effects of EPC-derived exosomal miRNAs. Thirdly, the role of SP1 and ELF5 in the phenotypic changes of fibroblasts to endothelial cells and angiogenesis of HCFs is still unclear in this study, and future studies may perform genome-wide expression analysis to elucidate the underlying molecular mechanisms. Fourthly, the neovascularization has been not determined by IHC in the *in vivo* studies, which may be considered in our future studies.

## Conclusion

In summary, our study revealed that miR-1246 and miR-1290 in EPC-derived exosomes are both beneficial for *in vitro* and *in vivo* angiogenesis in MI, and these improvements may be associated with amelioration of cardiac injury and cardiac fibrosis after MI.

## Data Availability Statement

The original contributions presented in the study are included in the article/Supplementary Material, further inquiries can be directed to the corresponding author/s.

## Ethics Statement

The animal study was reviewed and approved by the all the *in vivo* experiments were performed in accordance with a protocol approved by the Research Ethic Committee of Fuwai Hospital.

## Author Contributions

XK, YH, LC, and ZF performed the study and analyzed the experimental data. WC, SY, and RY performed the statistical analysis. JX, JG, and DZ performed the bioinformatics analysis. XK and YH designed the study and wrote the manuscript. All authors contributed to the article and approved the submitted version.

## Conflict of Interest

The authors declare that the research was conducted in the absence of any commercial or financial relationships that could be construed as a potential conflict of interest.

## Footnotes

1 https://imagej.nih.gov/ij/

## Supplementary Material

Supplementary Figure 1 Exosome significantly downregulated ACAT2, and upregulated CD31, VE-Cadherin and VEGFR2 in HCFs. Quantitative analysis of ACTA2, CD31, VE-Cadherin (VE-Cad) and VEGFR2 in HCFs after treatment with exosomes based on the immunofluorescence results. *N* = 3. ^∗∗^*P* < 0.01 vs. c ontrol group. Exo, exosomes.

Supplementary Figure 2 MiR-1246 and miR-1290 markedly affected angiogenesis induced by exosomes from EPCs. HCFs were addressed with exosomes from parent EPCs (Con/Exo), exosomes from EPCs transfected with NC (NC/Exo), inhibitor-1/Exo, Mimics-1/Exo, inhibitor-2/Exo or Mimics-2/Exo. **(A)** α-SMA, CD31, VE-Cad and VEGFR2 expressions were quantitatively analyzed in line with the results of immunofluorescence staining. **(B)** Relative Dil-ac-LDL intensity was calculated according to the result of DiL-Ac-LDL staining. *N* = 3. ^∗^*P* < 0.05, ^∗∗^*P* < 0.01 vs. NC/Exo group. Exo, exosomes; Mimics-1, miR-1246 mimics; Mimics-2, miR-1290 mimics; Inhibitor-1, miR-1246 inhibitors; Inhibitor-2, miR-1290 inhibitors.

Supplementary Figure 3 MiR-1246 and miR-1290 significantly upregulated ELF5 and SP1 in HCFs induced by exosomes. The immunofluorescence staining results of ELF5 and SP1 expression were quantitatively calculated in HCFs, which were treated with Con/Exo, NC/Exo, inhibitor-1/Exo, Mimics-1/Exo, minhibitor-2/Exo or Mimics-2/Exo. *N* = 3. ^∗∗^*P* < 0.01 vs. NC/Exo group. Exo, exosomes; Mimics-1, miR-1246 mimics; Mimics-2, miR-1290 mimics; Inhibitor-1, miR-1246 inhibitors; Inhibitor-2, miR-1290 inhibitors.

Supplementary Figure 4 Agarose gel electrophoresis shows the PCR results from each CHIP assay. **(A)** ELF5: 1, marker D2000; 2, input; 3, IgG; 4, NC; 5, mimics-1/Exo; **(B)** SP1: 1, marker D2000; 2, input; 3, IgG; 4, NC; 5, mimics-2/Exo.

Supplementary Figure 5 The impacts of miR-1246 and miR-1290 in exosomes on ELF5, SP1, α-SMA and CD31 expressions in in rats with myocardial infarction. After administration with exosomes from EPCs transfected with NC/Exo, Mimics-1/Exo or Mimics-2/Exo, IHC assay was applied to examine ELF5, SP1, α-SMA and CD31 expressions. The positive area of ELF5, SP1, α-SMA and CD31 were quantitatively analyzed. *N* = 10. ^∗∗^*P* < 0.01 vs. Sham group; ^##^*P* < 0.01 vs. model group. Exo, exosomes; Mimics-1, miR-1246 mimics; Mimics-2, miR-1290 mimics.

## Abbreviations

MI: myocardial infarction
EPC: endothelial progenitor cells
CF: cardiac fibroblast
EC: endothelial cells
ELF5: E74 like ETS transcription factor 5
CAD: coronary artery disease
IHD: ischemic heart disease
MEndT: mesenchymal–endothelial transition
HMGB1: high-mobility group box 1 protein B1
miRNA: microRNA
UTR: untranslated region
SD: Sprague-Dawley
ECG: electrocardiogram
TTC: 2,3,5-triphenyltetrazolium
H&E: hematoxylin abd eosin
EGM: endothelial cell growth medium
DiL-Ac-LDL: 1,1-dioctadecyl-3,3,3,3-tetramethylindocarbocyanine perchlorate
FITC-UEA-l: fluorescein isothiocyanate-Ulex europaeus agglutinin-1
qRT-PCR: quantitative reverse transcription PCR
ELISA: enzyme-linked immunosorbent assay
ANOVA: one-way analysis of variance
SEM: standard error of mean
PB: peripheral blood.
