# Supplementary figures and images for: EPC-Derived Exosomal miR-1246 and miR-1290 Regulate Phenotypic Changes of Fibroblasts to Endothelial Cells to Exert Protective Effects on Myocardial Infarction by Targeting ELF5 and SP1
Source: Front Cell Dev Biol. 2021 May 13;9:647763. doi: 10.3389/fcell.2021.647763 (PMC8155602; doi:10.3389/fcell.2021.647763)

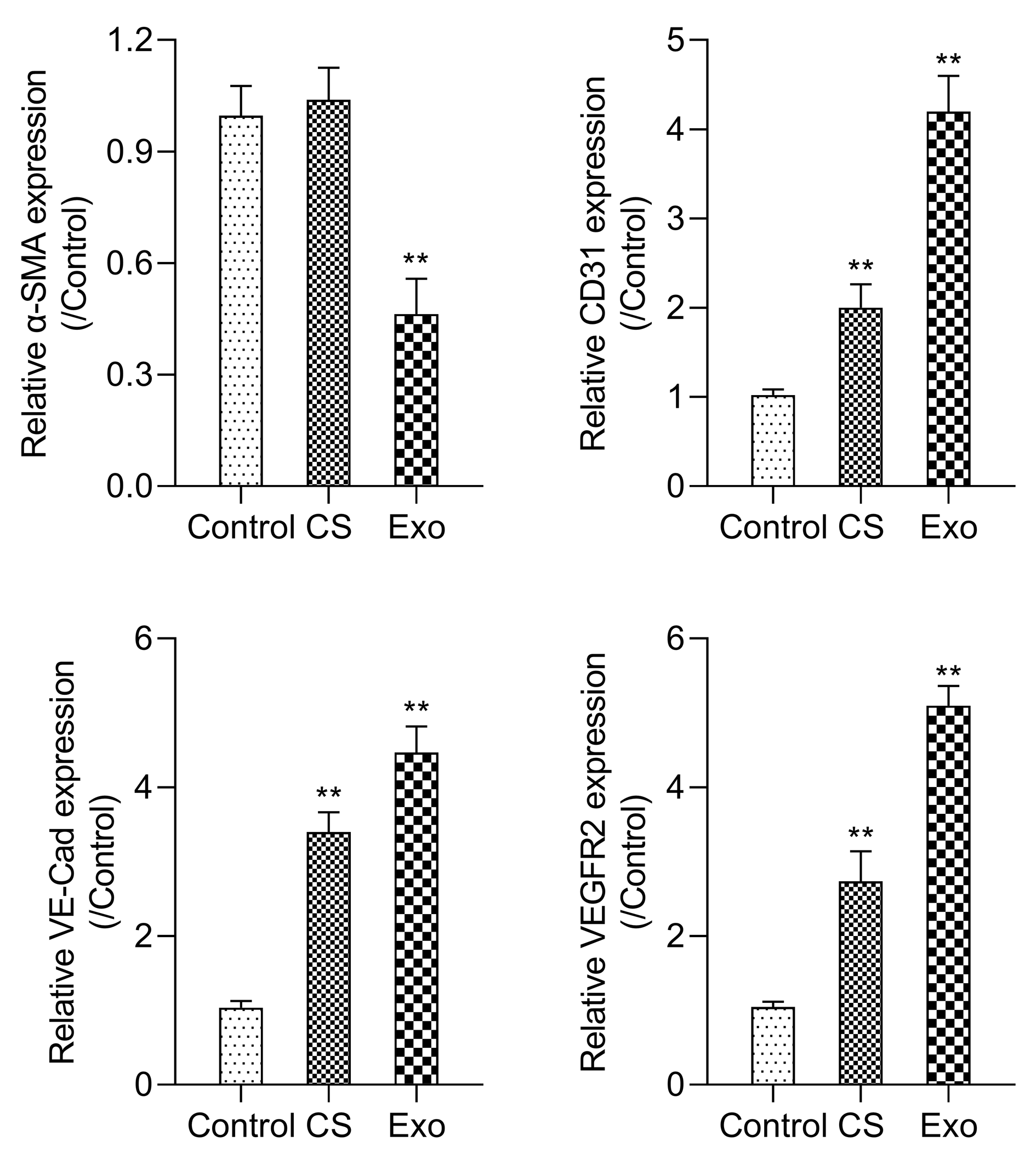

Supplement: Supplementary Figure 1 — Exosome significantly downregulated ACAT2, and upregulated CD31, VE-Cadherin and VEGFR2 in HCFs. Quantitative analysis of ACTA2, CD31, VE-Cadherin (VE-Cad) and VEGFR2 in HCFs after treatment with exosomes based on the immunofluorescence results. N = 3. ∗∗P < 0.01 vs. c ontrol group. Exo, exosomes. [file Image_1.tif]

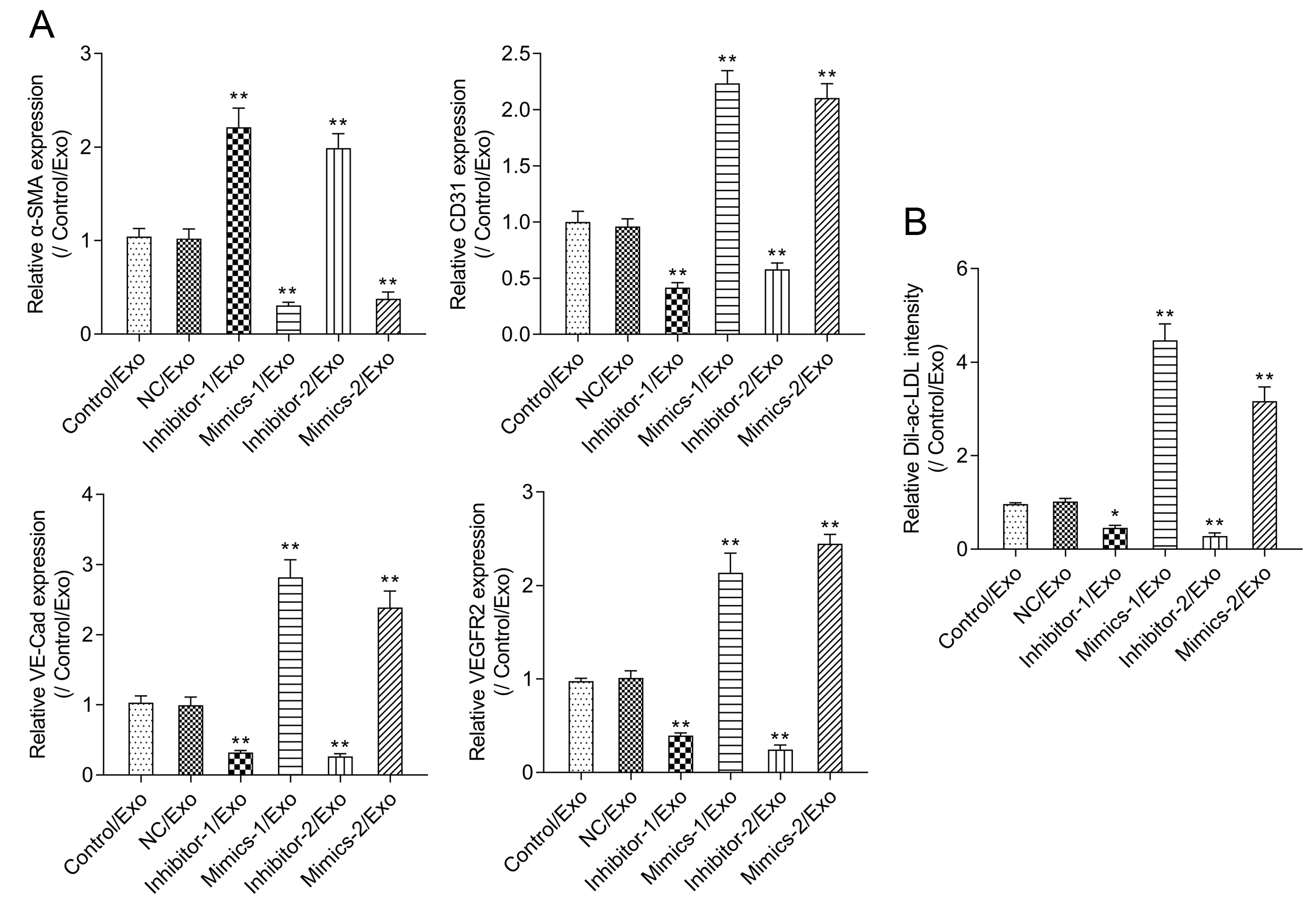

Supplement: Supplementary Figure 2 — MiR-1246 and miR-1290 markedly affected angiogenesis induced by exosomes from EPCs. HCFs were addressed with exosomes from parent EPCs (Con/Exo), exosomes from EPCs transfected with NC (NC/Exo), inhibitor-1/Exo, Mimics-1/Exo, inhibitor-2/Exo or Mimics-2/Exo. (A) α-SMA, CD31, VE-Cad and VEGFR2 expressions were quantitatively analyzed in line with the results of immunofluorescence staining. (B) Relative Dil-ac-LDL intensity was calculated according to the result of DiL-Ac-LDL staining. N = 3. ∗P < 0.05, ∗∗P < 0.01 vs. NC/Exo group. Exo, exosomes; Mimics-1, miR-1246 mimics; Mimics-2, miR-1290 mimics; Inhibitor-1, miR-1246 inhibitors; Inhibitor-2, miR-1290 inhibitors. [file Image_2.tif]

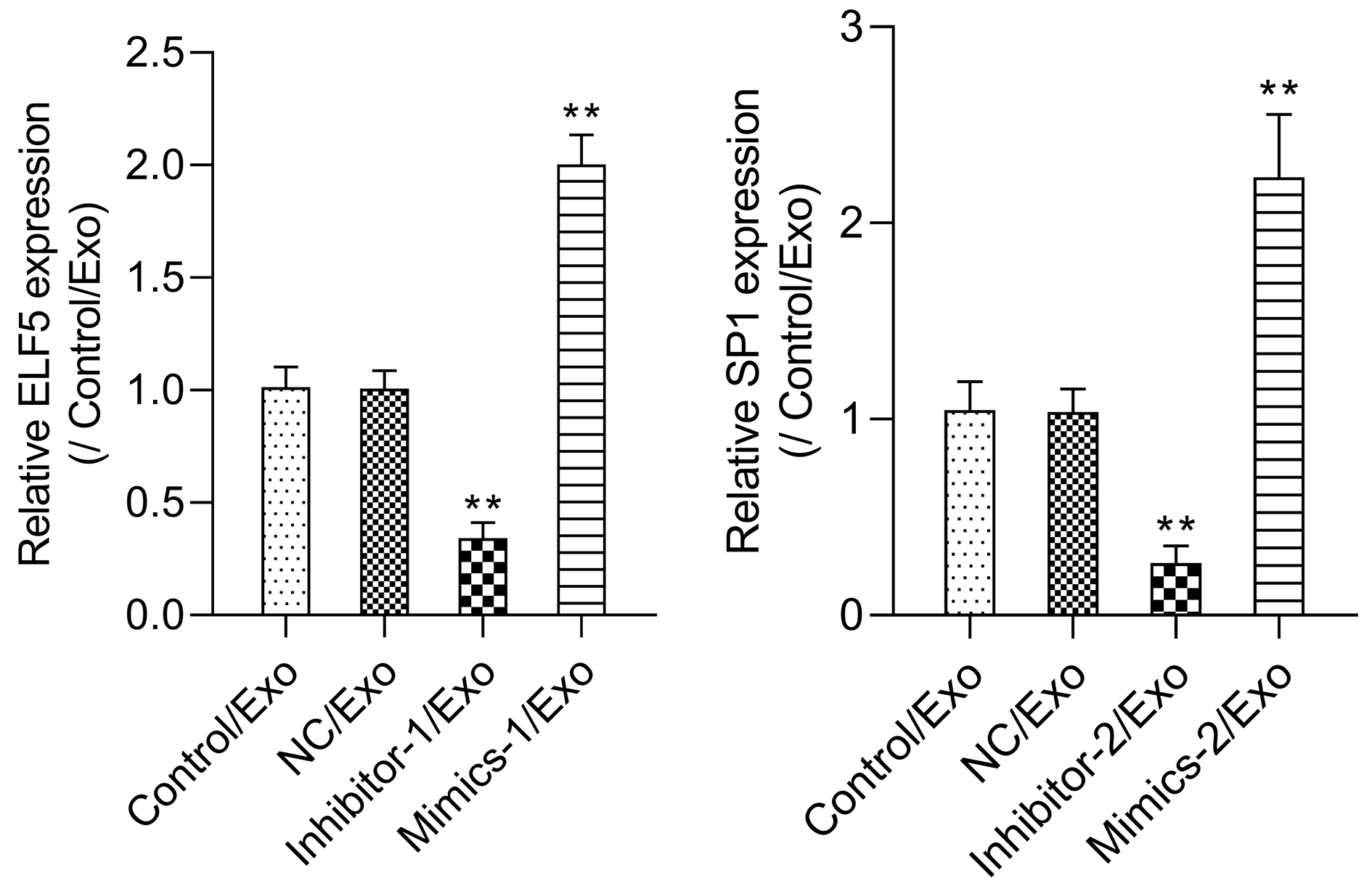

Supplement: Supplementary Figure 3 — MiR-1246 and miR-1290 significantly upregulated ELF5 and SP1 in HCFs induced by exosomes. The immunofluorescence staining results of ELF5 and SP1 expression were quantitatively calculated in HCFs, which were treated with Con/Exo, NC/Exo, inhibitor-1/Exo, Mimics-1/Exo, minhibitor-2/Exo or Mimics-2/Exo. N = 3. ∗∗P < 0.01 vs. NC/Exo group. Exo, exosomes; Mimics-1, miR-1246 mimics; Mimics-2, miR-1290 mimics; Inhibitor-1, miR-1246 inhibitors; Inhibitor-2, miR-1290 inhibitors. [file Image_3.tif]

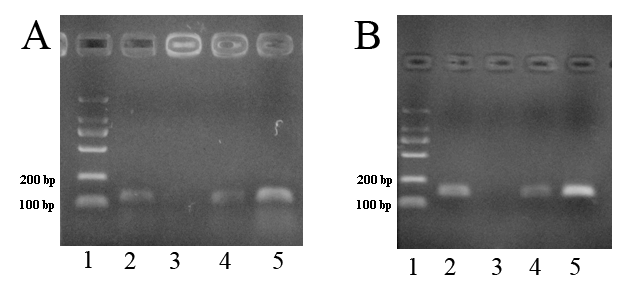

Supplement: Supplementary Figure 4 — Agarose gel electrophoresis shows the PCR results from each CHIP assay. (A) ELF5: 1, marker D2000; 2, input; 3, IgG; 4, NC; 5, mimics-1/Exo; (B) SP1: 1, marker D2000; 2, input; 3, IgG; 4, NC; 5, mimics-2/Exo. [file Image_4.tif]

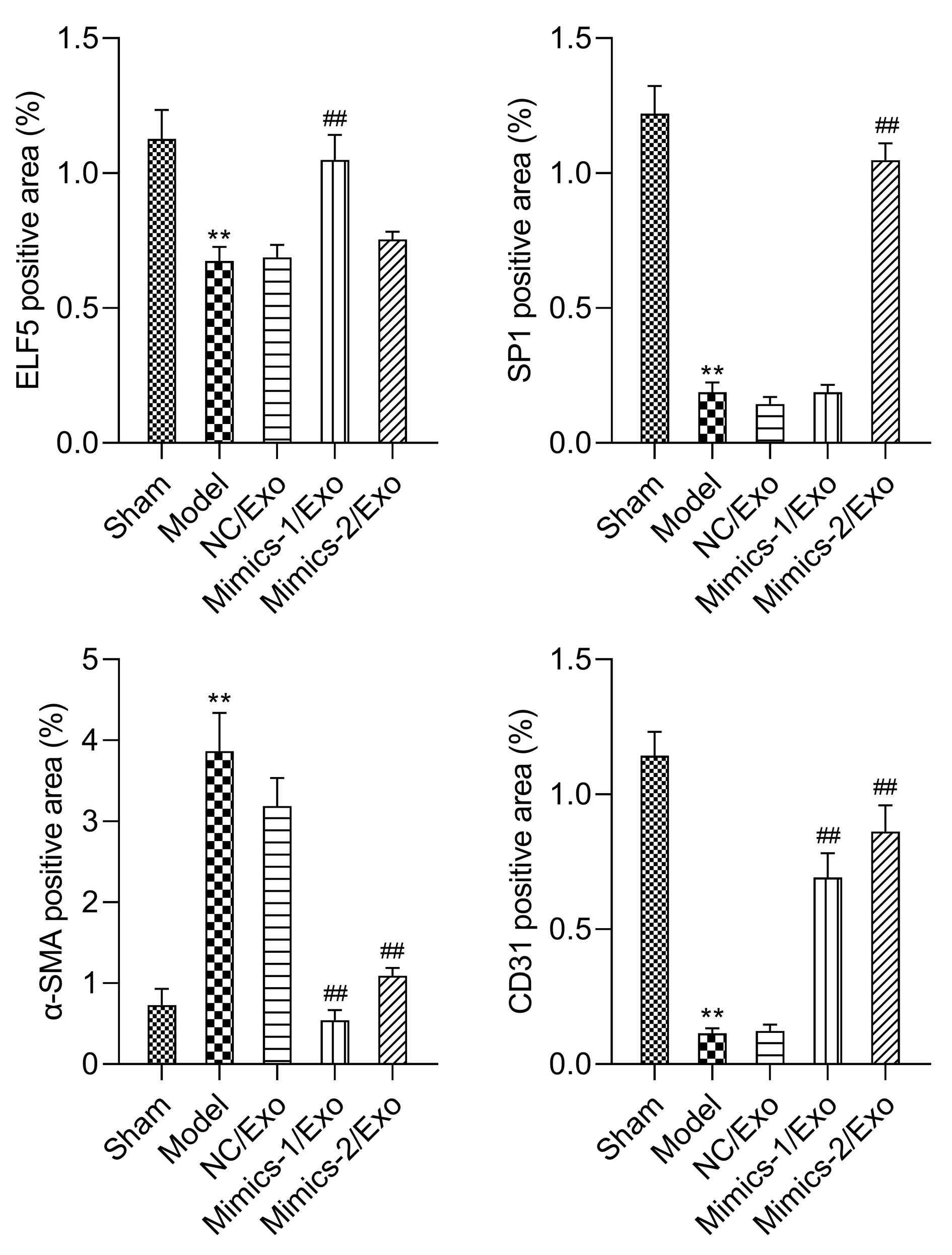

Supplement: Supplementary Figure 5 — The impacts of miR-1246 and miR-1290 in exosomes on ELF5, SP1, α-SMA and CD31 expressions in in rats with myocardial infarction. After administration with exosomes from EPCs transfected with NC/Exo, Mimics-1/Exo or Mimics-2/Exo, IHC assay was applied to examine ELF5, SP1, α-SMA and CD31 expressions. The positive area of ELF5, SP1, α-SMA and CD31 were quantitatively analyzed. N = 10. ∗∗P < 0.01 vs. Sham group; ##P < 0.01 vs. model group. Exo, exosomes; Mimics-1, miR-1246 mimics; Mimics-2, miR-1290 mimics. [file Image_5.tif]
