# Supplementary material for: EPC-Derived Exosomal miR-1246 and miR-1290 Regulate Phenotypic Changes of Fibroblasts to Endothelial Cells to Exert Protective Effects on Myocardial Infarction by Targeting ELF5 and SP1
Source: Front Cell Dev Biol. 2021 May 13;9:647763. doi: 10.3389/fcell.2021.647763 (PMC8155602; doi:10.3389/fcell.2021.647763)
Supplement: Supplementary file 6 [file Table_1.DOCX]

**Table 1. Primers for quantitative real-time PCR.**

| Name | Primer sequence (5’-3’) |
| --- | --- |
| U6 F | CTCGCTTCGGCAGCACA |
| U6 R | AACGCTTCACGAATTTGCGT |
| All R | CTCAACTGGTGTCGTGGA |
| hsa_miR-1246 | AAUGGAUUUUUGGAGCAGG |
| hsa_miR-1246 RT | CTCAACTGGTGTCGTGGAGTCGGCAATTCAGTTGAGCCTGCTCC |
| hsa_miR-1246 F | ACACTCCAGCTGGGAATGGATTTTTGGAG |
| hsa_miR-1290 | UGGAUUUUUGGAUCAGGGA |
| hsa_miR-1290 RT | CTCAACTGGTGTCGTGGAGTCGGCAATTCAGTTGAGTCCCTGAT |
| hsa_miR-1290 F | ACACTCCAGCTGGGTGGATTTTTGGATCA |
| hsa_miR-375 | UUUGUUCGUUCGGCUCGCGUGA |
| hsa_miR-375 RT | CTCAACTGGTGTCGTGGAGTCGGCAATTCAGTTGAGTCACGCGA |
| hsa_miR-375 F | ACACTCCAGCTGGGTTTGTTCGTTCGGCTCGC |
| GAPDH F | TGTTCGTCATGGGTGTGAAC |
| GAPDH R | ATGGCATGGACTGTGGTCAT |
| CD31 F | AACAGTGTTGACATGAAGAGCC |
| CD31 R | TGTAAAACAGCACGTCATCCTT |
| KDR F | GGCCCAATAATCAGAGTGGCA |
| KDR R | CCAGTGTCATTTCCGATCACTTT |
| α-SMA F | CCTGTGTTGTGGTTTACACTGG |
| α-SMA R | GGGGGAATTATCTTTCCTGGTCC |
| VE-Cadherin F | GTCAAGGTCAACGTCTTGGAC |
| VE-Cadherin R | GTTTTATGAGAAGCGTACCAGGT |
| ELF5 F | TAGGGAACAAGGAATTTTTCGGG |
| ELF5 R | GTACACTAACCTTCGGTCAACC |
| SP1 F | TGGCAGCAGTACCAATGGC |
| SP1 R | CCAGGTAGTCCTGTCAGAACTT |
