# Supplementary material for: EPC-Derived Exosomal miR-1246 and miR-1290 Regulate Phenotypic Changes of Fibroblasts to Endothelial Cells to Exert Protective Effects on Myocardial Infarction by Targeting ELF5 and SP1
Source: Front Cell Dev Biol. 2021 May 13;9:647763. doi: 10.3389/fcell.2021.647763 (PMC8155602; doi:10.3389/fcell.2021.647763)
Supplement: Supplementary file 7 [file Table_2.DOCX]

**Table 2. The predicted binding sites between SP1/ELF5 and CD31**

| Model name | CD31 promoter region | | | |
| --- | --- | --- | --- | --- |
|  | Start | End | Strand | predicted site sequence |
| SP1 | 1756 | 1766 | 1 | CCTCCGCCTCC |
| ELF5 | 2567 | 2575 | 1 | TACTTCCTC |
