# Supplementary material for: EPC-Derived Exosomal miR-1246 and miR-1290 Regulate Phenotypic Changes of Fibroblasts to Endothelial Cells to Exert Protective Effects on Myocardial Infarction by Targeting ELF5 and SP1
Source: Front Cell Dev Biol. 2021 May 13;9:647763. doi: 10.3389/fcell.2021.647763 (PMC8155602; doi:10.3389/fcell.2021.647763)
Supplement: Supplementary file 8 [file Table_3.DOCX]

**Table 3**. The related indicators of cardiac function in echocardiography.

| **Indicators** | **HR** | **LVESd (mm)** | **LVEDd (mm)** | **Vs (uL)** | **Vd (uL)** | **SV (uL)** | **LVEF (％)** | **FS (％)** | **CO (mL/min)** | **LVPWT (mm)** | **LVAWT (mm)** |
| --- | --- | --- | --- | --- | --- | --- | --- | --- | --- | --- | --- |
| Sham | 354±11.53 | 3.7±0.18 | 6.14±0.32 | 101.06±4.85 | 246.02±8.42 | 146.16±5.45 | 74.74±1.48 | 35.48±1.2 | 50.79±2 | 2.23±0.09 | 2.07±0.11 |
| Model | 281.33±9.61** | 5.38±0.22** | 6.82±0.46 | 98.61±6.58 | 211.69±5.96** | 115.91±3.42** | 51.17±2.07** | 26.85±2.19** | 34.01±2.17** | 2.17±0.08 | 1.92±0.1 |
| NC-Exo | 303.67±12.7 | 4.89±0.21 | 6.62±0.38 | 96.47±2.94 | 213.3±8.42 | 120.47±4.99 | 53.44±2.93 | 28.76±2 | 37.28±1.86 | 2.34±0.18 | 2.1±0.13 |
| Mimics-1/Exo | 323.67±3.21^##^ | 4.54±0.24^##^ | 6.71±0.3 | 100.17±2.5 | 229.77±2.1^#^ | 131.54±3.03^##^ | 60.45±2.07^##^ | 32.58±0.95^##^ | 43.74±3.29^##^ | 2.27±0.07 | 1.96±0.09 |
| Mimics-2/Exo | 327.67±11.02^##^ | 4.12±0.28^##^ | 6.62±0.27 | 97.94±3.47 | 237.57±4.53^##^ | 136.86±2.35 | 65.17±2.54^##^ | 34.31±0.79^##^ | 45.39±2.55^##^ | 2.15±0.07 | 2.07±0.08 |

***P* < 0.01 vs. sham group, #*P* < 0.05, ##*P* < 0.01 vs. model group. Exo, exosomes; Mimics-1, miR-1246 mimics; Mimics-2, miR-1290 mimics; Inhibitor-1, miR-1246 inhibitors; Inhibitor-2, miR-1290 inhibitors; HR, heart rate; LVESd, left ventricular end systolic diameter; LVEDd, left ventricular end-diastolic diameter; Vs, systolic left ventricular volume; Vd, diastolic left ventricular volume; SV, stroke volume; LVEF, left ventricular ejection fraction; FS, fractional shortening; CO, cardiac output; LVPWT, left ventricular posterior wall thickness; LVAWT, left ventricular diastolic anterior wall thickness.
